# Bentall procedure 39 years after implantation of a Starr-Edwards Aortic Caged- Ball-Valve Prosthesis

**DOI:** 10.1186/1749-8090-5-12

**Published:** 2010-03-18

**Authors:** Jan D Schmitto, Philipp Ortmann, Aron F Popov, Kasim O Coskun, Hanna Schotola, Martin Friedrich, Christoph H Wiese, Christian Sohns, Suyog A Mokashi, Vassilios N Didilis, Friedrich A Schoendube

**Affiliations:** 1 Department of Thoracic-, Cardiac- and Vascular Surgery, Georg-August-University of Goettingen, Goettingen, Germany; 2 Division of Cardiac Surgery, Brigham and Women's Hospital, Harvard Medical School, Boston, MA, USA; 3 Department of Anesthesiology, Georg-August-University of Goettingen, Goettingen, Germany; 4 Department of Cardiology and Pneumology, Georg-August-University of Goettingen, Goettingen, Germany

## Abstract

We report a case of a male patient who received an implantation of a Starr-Edwards-caged-ball-valve-prosthesis in 1967. The surgery and postoperative course were without complications and the patient recovered well after the operation. For the next four decades, the patient remained asymptomatic - no restrictions on his lifestyle and without any complications. In 2006, 39 years after the initial operation, we performed a Bentall-Procedure to treat an aortic ascendens aneurysm with diameters of 6.0 × 6.5 cm: we explanted the old Starr-Edwards-aortic-caged-ball-valve-prosthesis and replaced the ascending aorta with a 29 mm St.Jude Medical aortic-valve-composite-graft and re-implanted the coronary arteries.

This case represents the longest time period between Starr-Edwards-caged-ball-valve-prothesis-implantation and Bentall-reoperation, thereby confirming the excellent durability of this valve.

## Case report

**Figure 1:**
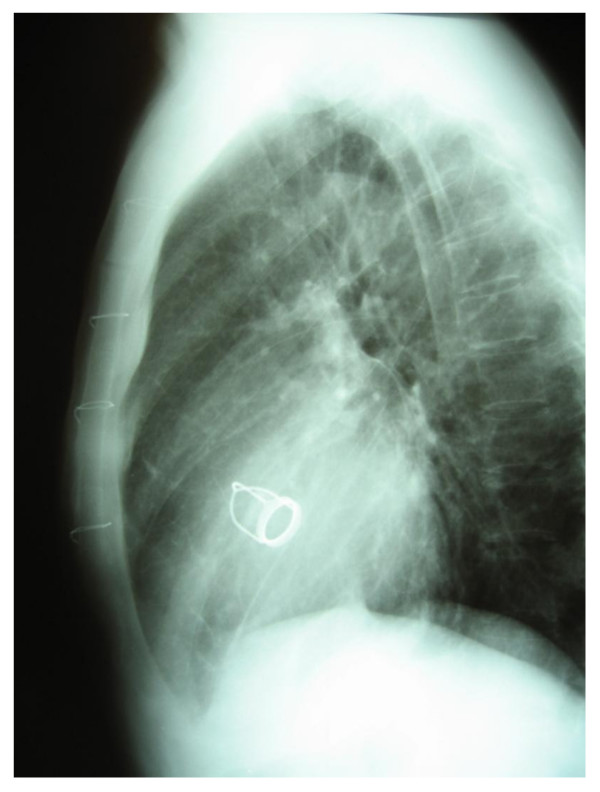
**Pre-operative chest X-ray with the Starr-Edwards-caged-ball-valve in aortic position**.

In 1967, a 23-year-old male presented with severe aortic valve regurgitation. Given the symptoms were refractory to medical management, the decision was made to replace the dysfunctional aortic valve. At that time, 39 years ago, standard care was to implant a Starr-Edwards-caged-ball-valve-prosthesis in aortic position (Figure 1). The surgery and post-operative course were without complications and the patient recovered well after the operation. However, four years later, the ball was damaged and contained thromboembolic formations. Therefore, the decision was made to replace the elastic ball with a structurally similar silicone ball. For the next four decades, the patient remained asymptomatic - no restrictions on his lifestyle and without any complications.

In January 2006, 39 years after the initial operation, the patient became increasingly symptomatic for six weeks - progressive dyspnea and chest pain (angina-pectoris according to CCS III). Myocardial scintigraphy and coronary angiography were performed and revealed diffuse, but insignificant atherosclerosis (possibly explaining his acute deterioration). An aortic angiogram revealed high grade post-valvular aneurysm of the ascending aorta. This was also proven on computed tomography of the thorax: the dimensions of the aortic aneurysm were 6.0 × 6.5 cm, with normal physiological diameters distally, with no evidence of an aortic dissection. Additionally, echocardiography demonstrated: middle-grade dilatation of the left atrium, high-grade dilatation of the left ventricle and a left ventricular ejection fraction (EF) severely reduced to 30%. The functionality of the prosthesis presented borderline values, but regular flow.

**Figure 2:**
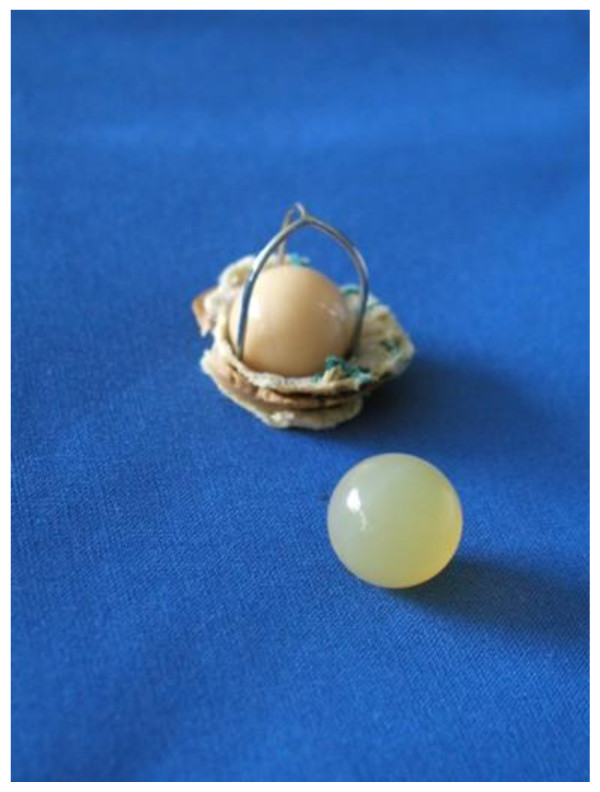
**Explanted Starr-Edwards-caged-ball-valve 39 years after implantation**.

**Figure 3:**
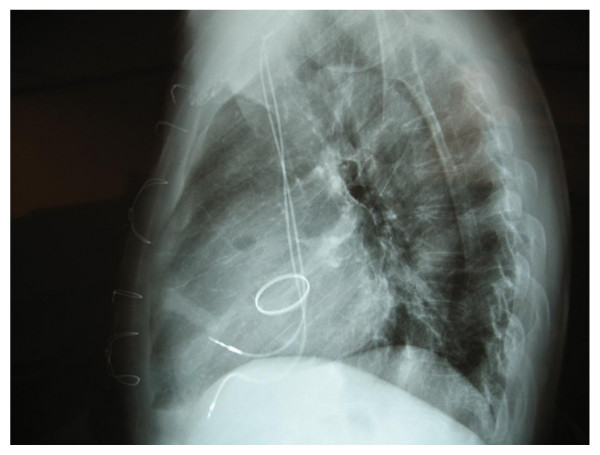
**Post-operative chest X-ray after successfully performed Bentall- and Pacemaker-implantation**.

Operative intervention was once again indicated. In February, we decided to perform a Bentall-Procedure: therefore, we explanted the 39-year-old Starr-Edwards-aortic-caged-ball-valve-prosthesis (Figure 2) and replaced the ascending aorta with a 29 mm St. Jude Medical aortic-valve-composite-graft and re-implanted the coronary arteries. Postoperatively, the patient developed AV-block III°, therefore a DDD-pacemaker was implanted four days later (Figure 3). The remainder of the postoperative course was uneventful and he was discharged home on postoperative day 12 with stable hemodynamics. On histological analysis of the aortic wall, a large calcification, bulky mucoid degeneration and destruction of elastic fibres was found.

Of note, this coincidentally is the latest Bentall-procedure after 39 years of survival of a Starr-Edwards-valve implantation found in the literature [1].

## Discussion

The Starr-Edwards caged-ball-valve prosthesis (Edwards Lifesciences, Nyon, Switzerland) was implanted routinely for many years in heart centers worldwide. By 1967, nearly 2000 Starr-Edwards-valves had been implanted [2] - establishing the caged-ball-valve prosthesis as the standard surgical treatment for aortic and mitral valve disease. Due to the fact that the Starr-Edwards valves showed a high ventriculo-aortic outflow pressure gradient and also a higher incidence of poststenotic dilations of the ascending aorta in long-term follow-up, newer models had to be developed to increase the valve's hemodynamics. Therefore, new valve prostheses were manufactured using several different materials, including: pericardium, fasciae latae, and dura mater. In the 1960s, Binet and colleagues began to develop tissue valves [2]. Then in 1964, Duran and Gunning (Oxford, England) replaced an aortic valve in a patient using a xenograft porcine aortic valve [3]. The early results of the Starr-Edwards-valve were good [1], however after a few years these patients developed signs of chronic heart failure, due to the poor hemodynamics of the valve. In addition, the caged-ball-valves showed a higher incidence of thrombembolic complications compared to the newer valve developments. However, there is one large series reporting on 2,247 aortic valve replacements with Starr-Edwards prostheses implanted between 1960 and 1997 [1]. In this study, Kaplan-Meier (KM) survival at 10 years was 53% for Aortic Valve Replacement, 23% at 20 years, 8% at 30 years and 4% at 40 years. Even in this study no Bentall operation after such a long time period was presented. We therefore present the longest time period between Starr-Edwards-caged-ball-valve-prothesis implantation and Bentall reoperation of a long-term survivor, thereby confirming the excellent durability of this valve.

## Competing interests

The authors declare that they have no competing interests.

## Authors' contributions

All authors read and approved the final manuscript.

## Consent

Written informed consent was obtained from the patient for publication of this case report and accompanying images. A copy of the written consent is available for review by the Editor-in-Chief of this journal.
